# To build a better VAD: What is needed to make MCS truly competitive with cardiac transplantation for long-term outcomes?

**DOI:** 10.1016/j.jhlto.2025.100325

**Published:** 2025-06-20

**Authors:** Jennifer A. Cowger, Evgenij Potapov

**Affiliations:** a Department of Cardiovascular Medicine, Henry Ford Health, Michigan State University and Wayne State University, Detroit, MI; b Deutsches Herzzentrum der Charité, Department of Cardiothoracic and Vascular Surgery, Augustenburger Platz 1, 13353 Berlin, Germany; c Charité – Universitätsmedizin Berlin, corporate member of Freie Universität Berlin and Humboldt-Universität zu Berlin, Charitéplatz 1, 10117 Berlin, Germany; d German Centre for Cardiovascular Research, Partner Site Berlin, Berlin, Germany

**Keywords:** LVAD, Heart failure, Heart transplant, Outcomes, Survival, Adverse events

## Abstract

While average survival in patients undergoing durable left ventricular assist device (dLVAD) support now approximate 6 years, the application of therapy remains limited across the globe. Herein we highlight important deficiencies common to present technology and propose device innovations and field corrections that will be necessary to improve acceptance of dLVAD support by both patients and medical providers caring for patients with end-stage heart failure. Pragmatic trials will also be needed to help identify the therapeutic strategy (permanent dLVAD support vs heart transplant vs dLVAD as a bridge to transplant) that affords the best outcomes for a patient’s risk profile and total survival goals.

In the present era, management options for patients with chronic, end-stage heart failure (HF) with reduced ejection fraction (HFrEF) or recalcitrant cardiogenic shock include heart transplant (HT), durable mechanical circulatory support [MCS, most commonly in the form of durable left ventricular assist device support (dLVAD)], and hospice/palliative care. The application of each treatment is variably available and applied across the world. While hospice and palliative care are vital and necessary treatment options for the patient with end-stage HF, this review will focus on dLVAD and HT therapies, specifically with an aim to 1) compare and contrast the individual interventions as they relate to different patient populations and to 2) discuss advancements needed to ensure a steadfast evolution and acceptance of the therapies offered by our field.

## Outcomes with heart transplant and LVAD

**Figure 1:**
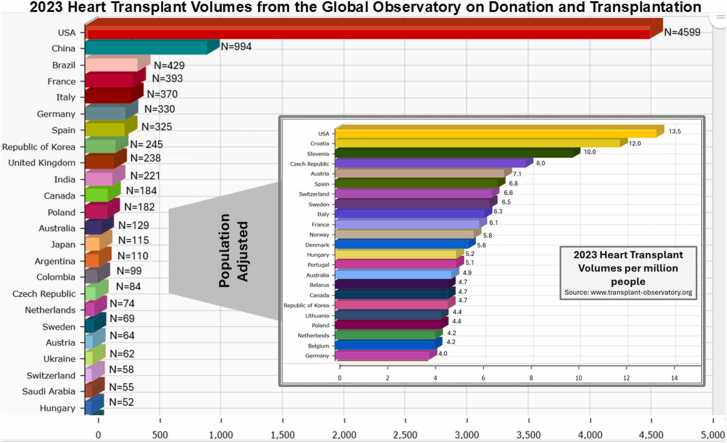
*Heart transplant volumes across the globe in the year 2023.* Heart transplant volumes for 2023 are presented for countries providing data to the global observatory on donation and transplantation. The inset shows the rate of heart of transplant, adjusted per million people. Data available at: www.transplant-observatory.org.

Statistics suggest that over 64 million persons are living with HF across the globe (age standardized prevalence rate of 71.45 per 100,000) with an attributable mortality rate of 4.95 per 100,000 persons.^1^ HT is felt by many to represent the field’s “gold standard” treatment for eligible patients with end-stage HF. Figure 1 shows the frequency of HT performed by several top transplanting countries across the world in 2023, while the figure inset shows the HT rate per million inhabitants. While the U.S. had the highest numbers of HTs overall, it is astounding to realize that only 13.5 heart transplants occur in the U.S. per million inhabitants.^1^ Overall, access to HT is highly variable across the globe, and the gift of HT is rare.

In the 2023 Thoracic Organ Transplant Registry from the International Society of Heart and Lung Transplantation, of the 97,140 patients undergoing HT between 1992 and 2017, freedom from death or retransplant was 50% at ∼11 years, with a mean survival of 12-13 years for those undergoing HT after 2001.^2^ Survival following HT, when compared to other interventions undertaken for patients with severe disease (e.g., stage 1-2 breast cancer, prostate cancer, renal cell carcinoma),^3^ is quite poor, especially for young persons seeking extended survival gains. The field has only witnessed an approximate 3 year gain in average absolute HT survival (from 10 years to 13 years) over the last 20 years. Research and development in HT has largely focused in the recent era on increasing organ access (e.g., donation after circulator death, Hepatitis C heart donors, and organ preservation with warm vs cold storage technologies) or noninvasive monitoring after HT, not on mechanisms for impacting the long-term survival trajectory after HT.

The field of dLVAD, in contrast, has enjoyed an early, rapid technological evolution that has led to improved survival and a reduction in adverse events (AEs). Compared with average survivals of 1 and 4.5 years with application of first-generation^4^ and second generation technologies,^5^, ^6^ respectively, contemporary average survival after third generation HeartMate 3 (HM3, Abbott, Illinois, US) dLVAD surgery in both clinical trial and large registries approximates 6 years in the U.S., Canada, and Europe.^7^, ^8^, ^9^ For the first time in the field’s history, dLVAD support offers a reasonable survival option compared to that of HT in the first 3 years (survival age <50: 82% and age 50-59: 75% for dLVAD vs 85% for HT).^10^, ^11^ The field’s excitement about dLVAD support for the management of end-stage HF was initially palpable, spurring increased device utilization. In some parts of the world, the approval of destination therapy (DT) dLVAD support enabled more persons with advanced HF to have to access to life saving therapy when HT was not feasible. In the U.S., for example, the HM3 was approved for short-term (i.e., bridge to transplant) support in 2017 and DT in 2018. The numbers of Food and Drug Administration-approved dLVAD implants reported to the Society of Thoracic Surgeons’ Intermacs dLVAD registry increased from 2621 devices in 2013, peaking at 3201 patients in 2019.^5^, ^12^ In Germany, device utilization also increased, peaking at 1003 in the year 2017.^13^

**Figure 2:**
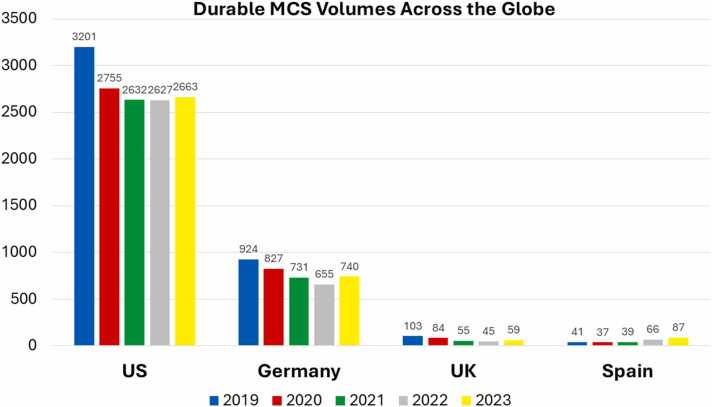
*Durable mechanical circulatory support volume (2019-2023).* Annual implant volumes of durable mechanical circulatory support devices (MCS) are presented for the United States (US), Germany, UK (United Kingdom), and Spain for years 2019 to 2023. Volumes have not increased despite improvement in contemporary durable left ventricular assist technology and associated patient outcomes.

Incongruously, however, despite the clinically significant gains in survival and AE profiles with HM3 technology, volumes of dLVADs implants in several regions of the world have started to plateau or even decline. Figure 2 shows dLVAD volumes between 2016 and 2023 for the U.S.,^10^ Germany,^13^ Spain,^14^ and the UK.^15^ In the US and Germany, device utilization has declined by 25% when compared to peak volumes.^10^, 13.

## The key obstacles to the application of LVAD therapy

There are several important obstacles impeding expanded application of dLVAD therapy, which will require urgent and collective engagement from industry, physicians, patients, health care payers, and regulatory agencies so that the evolution of the field—which is starting to stall—is re-invigorated and feasible.

### Field opportunities: Acknowledging and improving the LVAD patient’s experience

At the very center of this collective effort is the engagement of patients into dLVAD system design and health related quality of life (hrQOL) assessment. Bill Gates, while leading a company in a different industry (Microsoft, Inc.), summarized our field’s needs quite apropos: “The advance of technology is based on making it fit in so that you don’t really even notice it, so it’s part of everyday life.” For dLVAD systems to be embraced by more patients and recommended by referring clinicians and advanced HF specialists, the burdens of support must be reduced, and the technology must be hidden- or at the very least- be forgettable. To this end, the evolution of current- and the introduction of next generation devices- must demonstrate more extensive incorporation of human factors engineering and research. Human factors research combine findings from device engineering, psychology, and cardiology/surgical clinical science into device design. While human factors testing is already a Food and Drug Administration requirement for dLVADs under study, the focus is on key safety factors that may lead to patient errors, device or patient harm, and/or device recalls. There is, however, a need to expand human factors research to optimize device design for special populations (e.g., pediatrics, those with obesity, arthritis, or hearing or visual impairments), allowing better integration of the dLVAD and its peripheral components into a patient’s life, with the potential to improve hrQOL.

Widespread application of dLVAD support is also obstructed by the permanent lifestyle changes that patients and care givers must make to ensure there is no damage to the device peripherals (e.g., avoiding water or electromechanical interference); there is uninterrupted system function (e.g., indubitable charge to batteries to ensure uninterrupted power); and the driveline is without trauma or signs of infection. The patient is permanently tethered to 7-10 pounds of equipment-awake or asleep. The psychological impact of the externalized components of the dLVAD support system also cannot be ignored. The batteries, controller, driveline, and audible beeps are a constant reminder to the patient of their illness, may be a source of negative body image,^16^, ^17^ and may contribute to anxiety and/or depression because they remind patients of their persistent dependence on technology for survival. From a hrQOL perspective, when alarms arise, there is presently no easy way to download data from a patient’s home and rapidly differentiate what could be a nuisance alarm (e.g., electrostatic discharge) from more critical pump dysfunction. Similarly, hospitalizations for nonLVAD related issues (e.g., hip surgery) usually require the patient to bypass the more convenient local hospital so a dLVAD trained team is present for device monitoring. Given the limited number of dLVAD centers in the world, travel for health care can be a very onerous for many patients and caregivers (and dLVAD team members), especially those who live in rural regions of the world or have limited resources. There is no hrQOL metric that presently can capture these inconveniences. We must begin to design devices for patients with a disease and not devices for the disease of HFrEF alone.

### Field opportunities: The physician perspective

While survival has markedly improved with present dLVAD technology, the AE profiles remain too high. Within 3 years of contemporary dLVAD implant, incident infection, stroke, and gastrointestinal bleeding occur in 30%, 10%, and 21% of patients, respectively.^10^ Persons with incident infection, gastrointestinal bleeding, and recrudescent HF after dLVAD tend to have recurrent events and worse long-term survival.^18^, ^19^ During chronic dLVAD support, readmission rates are 1.33 per patient year,^10^ burdens that are too high to allow for generalized, wide-spread expansion of dLVAD support across national health care systems.

#### Fully implantable left ventricular assist system

Broadly, we feel the most urgent innovations to facilitate dLVAD expansion and acceptance by more physicians and patients are related to dLVAD design. To address patient hrQOL and provider equipoise and to address the growing morbidity and mortality burdens from driveline infections, the dLVAD system must be internalized (termed fully implantable left ventricular assist system, FILVAS) with the capacity for rapid wireless data communication and pump management. The authors herein are not trivializing the technological revolution and capital investment that will be required to achieve these aims. While present dLVAD technology has been miniaturized, the energy consumption is far greater than smaller implantable technologies that run off internalized batteries. For example, the average power consumption of the HeartMate 3 is 3.0-6 W, while pacemakers consume 10-100 microwatts annually with a peak demand of only 100-200 microwatts. The greater the device’s power consumption, the larger the need for heat dissipation and the greater the risk for thermal injury to tissue. In systems that rely on coils to supply transcutaneous electricity systems (TETS), heat can also be generated by the coils, especially those that work at higher frequencies, and electromagnetic interference can complicate function.^20^ Other hurdles include the time required for the extensive testing needed to meet regulatory requirements and the notable economic requirements to support this aspect of technological advancement.

In addition to innovations in energy, the “brains” of the device must be fully internalized and easily accessible. There must be a capacity for data storage, and these data must be easy to obtain, allowing data downloads to occur quickly *and* from the comfort of a patient’s home. The data output must be informative to providers, and key data should be retrospective out 90-365 days, not the much shorter data time horizon provided by present technology. Ideally, providers will be able to make device program changes virtually and will have 24-7 support from an industry call center.

While the feats above may seem insurmountable, the history of the cardiovascular field, in general, should serve as encouragement for steadfastness. In the early 1950s, for example, Zoll pacemaker technologies were bulky, external devices (the size of a microwave oven) for which patients were tethered to an extension cord. Today, leadless pacemakers (Micra, Medtronic, Minnesota, US) the size of a vitamin capsule with 17 years of life are available. Similar field evolutionary success has also been noted in transcatheter valvular interventions. While patience is challenging for providers who are used to making rapid choices and interventions, it is important to understand that the field of advanced HF has had experience with FILVAS (e.g., LionHeart, ABIOCOR)^21^ and we are not the only field that is seeking improved battery technology, wireless energy transfer, and/or improved wireless communications. Similar technologies are desired by the neuro-recovery industry and pain management, etc., supporting investment across disciplines, while the automotive industry has contributed greatly to the evolution of battery technology. The dLVAD industry is preparing for FILVAS. Corvion (Webster, Texas), for example, has a dLVAD under development that purports the capacity to provide 5 L of support with only 1.5 W of power consumption supplied via an implantable battery that has a 12 hours charge. Abbott has been partnering with Resonlink to develop a reliable TETS to support FILVAS,^22^ and there has been a notable uptick in research/collaboration by several companies (e.g., Minnetronix (Minnesota, US), Leviticus (Israel), FineHeart (France), BiVACOR (US)) examining both TET/coplanar energy transfer with some reporting first in human studies that are either under way or slated for the coming years.

#### Physiologic support

While dLVAD support can normalize resting cardiac output, devices that modulate flow during periods of exertion and sleep are needed. The pulsatility algorithm of HM3 attempts to impart some flow pulsatility through pump speed increases and decrements, but the changes in pulse pressure are on average very small and speed modulation is not timed to aortic valve opening and/or heart rate. The lack of synchronized speed augmentation during high physiologic demand is in part why presently approved technologies have never been shown to impart notable increases in patient peak oxygen consumption.^23^ During periods of rest, de-escalation of flow may be equally favorable, reducing power consumption (extend battery life) and niduses for aortic insufficiency development. In patients who will tolerate lower nocturnal speeds, speed reduction may promote aortic valve opening and reduce exposure of the root side of the aortic valve to continuous high shear stress and strain that promote valve degeneration, fusion, insufficiency, and thrombosis.^24^, ^25^ The field looks forward to clinical data from the Corwave (Clichy, France) device, which boasts high fidelity pulsatility and physiologic flow modulation.

#### One size does not fit all

The future generation of dLVAD should also provide surgeons with the ability to select from variable pump flow capacities and inflow depths. Additional pump sizing options are needed to enable a surgeon to select a pump that is designed to provide a specific range of indexed blood flow for a given patient’s needs, allowing pump flows to fall within the range for which it was designed and recommended per device instructions for use. This would be beneficial for pediatric patients, adult persons of smaller body size, and adult persons of very large body size. Additionally, pumps should be designed with an option for different inflow lengths for a given pump housing, different implantation locations (ventricular or atrial), as well as for different heart sides (left or right). Until FILVAS is developed, different driveline lengths should also be designed. The former may facilitate improved ventricular washing and may optimize inflow interfacing with the ventricle walls and papillary muscles; the later would enable selection of a driveline length that accounts for a patient’s larger or smaller abdominal habitus.

Finally, technologies addressing persons with biventricular failure warrant ongoing investigation. The field awaits data from BiVACOR (California, US) and the newly engineered Syncardia Emperor total heart (Arizona, US) devices. While a small subset are in need, the total artificial heart options presently available for approved use are antiquated. A future where utilization of upfront total heart support (vs bailout bivad support) can be envisioned if devices with excellent long-term clinical outcomes are developed.

### Opportunities for the field overall going forward

**Figure 3:**
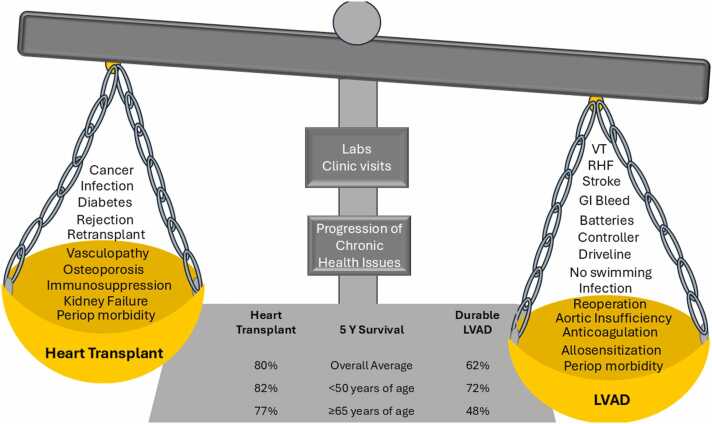
*Considerations for patients and clinicians when comparing heart transplant with durable left ventricular assist device support for management of end-stage heart failure.* Abbreviations: GI, gastrointestinal; LVAD, left ventricular assist device; RHF, right heart failure, VT, ventricular tachycardia.

While survival is a critical metric that guides therapeutic application, it is not the only metric (Figure 3). Within our field, we must begin to consider metrics other than survival when comparing HT to dLVAD. While HT is clearly the superior therapy when one looks at average long-term survival and hrQOL, an overly narrow focus on national HT volumes and posttransplant outcomes will 1) potentially yield biased benchmarks for establishing therapeutic success for end-stage HFrEF, 2) contribute to longer HT wait list times through reduced dLVAD utilization, and 3) will forget an entire population of patients who are not HT candidates. A comparison of survival following HT and dLVAD at 1 and 5 years is fraught with immortality bias.^26^ To adequately compare dLVAD with HT, one most compare the strategy of direct HT with that of dLVAD, the former of which must include the morbidity and mortality encountered on the HT wait list. For some patients, especially those over the age of 65 years, extended wait times on the HT list may reduce long-term survival probability due to increased wait list mortality and reduce survival after HT, especially when compared to outcomes in younger patients.^11^ When the strategy of HT is compared directly to contemporary dLVAD in persons over 65 year of age in the U.S., outcomes may not be statistically equivalent (2 years age ≥65 years: HT-22% died or delisted too ill vs LVAD mortality of 25% for ages 65-69 years and 29% for ages ≥70 years), but they also may not necessarily be viewed as inferior by society.^26^ The field and national health care systems need to determine a generally acceptable survival for persons with higher risk phenotypes, especially when donor hearts are of limited supply. In the U.S., a values prioritization survey is sent for open public comment and is used as guidance for organ prioritization during organ allocation changes when scientific data are limited. In the 2024 survey guiding development of a continuous heart allocation system, the U.S. public felt strongly that younger recipients should receive favor over older persons in heart organ allocation.^27^ In contrast to public sentiment, more persons over the age of 65 have been listed and transplanted in the U.S. in the last 5 years than in any prior era.^11^

Younger patients and those with other lower risk traits should have care plans focused on a strategy that achieves the most total years of life balanced within a patient’s personal view of hrQOL with each therapy. In the first 2-3 years of dLVAD support, for example, outcomes in persons <50 years of age on HM3 support are very respectable (70% at 5 years).^10^ If you add this survival to the subsequent survival following HT, total life years may be extended.

#### Expansion of competing temporary devices within the field of advanced heart failure

The impact of temporary mechanical support (tMCS) industry expansion and influence on patient management and dLVAD application must be acknowledged. While the field lacks adequate head to head data to support the benefit of one tMCS device versus another or versus inotropes in persons being considered for either HT or dLVAD, tMCS device utilization has soared across the globe. In parts of the world, tMCS expansion has also influenced policy; patients in the U.S., for example, with a low cardiac index and other risks managed with tMCS have been given preference for HT over those on dLVAD support. Given that most patients undergoing dLVAD implant were in some degree of cardiogenic shock prior to implant, this seems amnestic to the dLVAD patient’s prior treacherous journey and the deprioritization in UNOS has led to a marked reduction in clinical equipoise for the application of dLVAD support as a bridge to HT in the US. One must also consider the inherent and often unpredictable risks of dLVAD bridging, including allosensitization (de novo or from blood product exposure), stroke, infection, and perioperative complications (renal failure, prolonged respiratory failure with tracheostomy) that can impact a patient’s future transplant candidacy. Similarly, transplant must be considered in the context of its own serious AEs, including early operative events (primary graft failure, debilitation from long inpatient wait times) and long-term complications (e.g., coronary vasculopathy, increased propensity for cancer, infection, and renal failure) of immunosuppression. Overall, pragmatic trials are needed to better understand survival gains from tMCS according to specific patient risk groups (acuity, HF etiology, age, concomitant organ dysfunction) with a focus on the benefit of tMCS use in patients undergoing contemporary dLVAD vs. HT. This topic will become even more pertinent should a volume of patients enter the population on future dischargeable device technologies applied for extended (e.g., 6-12 months) tMCS (e.g., Abiomed, etc.). While data are needed, one could project that the combined benefit for extended tMCS and information gained from the burgeoning field of myocardial recovery may allow a clinically relevant subset of patients with advanced HF to achieve myocardial recovery, obviating the need for either transplant or durable LVAD.

#### Challenges within a niche and expensive field

While patient centric device designs with reduced AE rates are critical for expansion of device therapy for end-stage HFrEF, other opportunities exist to improve patient hrQOL. Warfarin use and associated dietary/drug interactions and the need for frequent lab monitoring add to dLVAD patient burdens. While pilot study data support the use of direct oral anticoagulants (e.g., apixaban) in patients on dLVAD support^28^, ^29^ the field needs adequately powered data and trial sponsorship to enable choice in anticoagulation regimen. However, with a low rate of clinical events after 90 days, who would support such a trial in a niche market that will require hundreds of patients for adequate powering? Similar challenges exist when examining the benefit of guideline directed medical therapy during dLVAD support.

Most importantly, the advanced HF field in general will be stagnated in growth and generalizability due to its expense and complexity. The costs associated with HT have soared in the last decade, for multifactorial reasons. While testing requirements vary across the world, the volume of tests required to assess and maintain candidacy on a HT list is vast. Care costs also need to account for utilization of one or more tMCS devices, complications encountered while waiting variable time on the HT wait list, and the time a bed is occupied due to high acuity wait listing status. In the present era, fees from organ procurement, utilization of organ perfusion devices or services, costs of fixed wing organ transport, and/or application of expensive pharmaceutical regimens for desensitization, hepatitis C donors, rejection monitoring, etc. are exorbitant. For dLVAD, the costs of the LVAS, its maintenance, the army of people trained for dLVAD care (VAD coordinators, anesthesiology, etc), regular lab testing, AEs, and readmissions cannot be ignored. This is compounded by the expense and slowness of device development and the regulatory requirements that must be met for device approval, taking over 10 years before devices achieve approval. Additionally, given the numbers of devices in various stages of development in a relative small field, venture capital investment runs the risk of dilution, and clinical trial design and enrollment may be hampered, impeding the success and timely market approval of ultimately impactful devices.

## Conclusion

The evolution of dLVAD technology has led to survival gains similar to that of HT at 2-3 years and survival averages now approach 6 years for the average patient and is over 7 years for those under age 50 at the time of device implant. While admirable, momentum within the dLVAD field has started to wane across the globe due to innovation and financial obstacles as well as influences favoring direct transplant. Going forward, the field needs to more carefully consider the survival goals of an individual patient and how the goals fit within a patient’s risk profile for HT or dLVAD and the health care structure that ultimately determines therapeutic access. Ultimately, a FILVAS will address several key issues that have impacted patient and field equipoise but will most certainly not resolve several critical AEs (e.g., bleeding, stroke, right HF) impacting patient outcome. Importantly, we as a field must require devices be designed for patients living with HFrEF, including care burdens and treatment specific hrQOL metrics in trial secondary endpoints.

## Declaration of Competing Interest

The authors declare the following financial interests/personal relationships, which may be considered as potential competing interests: Jennifer Cowger reports a relationship with Medtronic Inc. that includes: consulting or advisory. Jennifer Cowger reports a relationship with Abbott Laboratories Inc. that includes: consulting or advisory, speaking and lecture fees, and travel reimbursement. Jennifer Cowger reports a relationship with BrioHealth Solutions that includes: consulting or advisory. Evgenij Potapov reports a relationship with Abbott Laboratories Inc. that includes: consulting or advisory and speaking and lecture fees. Evgenij Potapov reports a relationship with AbioMed Inc. that includes: consulting or advisory and speaking and lecture fees. Evgenij Potapov reports a relationship with Medtronic Inc. that includes: consulting or advisory and speaking and lecture fees. Evgenij Potapov reports a relationship with Recovery Therapuetics that includes: consulting or advisory and speaking and lecture fees. Dr. Evgenij Potapov has received monies for Abbott related to proctoring. Dr. Cowger has served as an unpaid consultant for CorWave. If there are other authors, they declare that they have no known competing financial interests or personal relationships that could have appeared to influence the work reported in this paper.

## Acknowledgment

The authors are grateful for data provided on national dLVAD volumes from Manuel Gómez Bueno, MD (Spain), and Theo DeBy, PhD (UK).

## Financial support

None.
